# Lameness in Sheltered Cows and Its Association with Cow and Shelter Attributes

**DOI:** 10.3390/ani9060360

**Published:** 2019-06-16

**Authors:** Arvind Sharma, Clive J. C. Phillips

**Affiliations:** Centre for Animal Welfare and Ethics, School of Veterinary Science, The University of Queensland, Gatton Campus, Gatton QLD 4343, Australia; c.phillips@uq.edu.au

**Keywords:** cow shelters, lameness, risk factors, welfare assessment, indicators

## Abstract

**Simple Summary:**

The sheltering of cows in shelters is a traditional practice in India. Old, abandoned and unproductive dairy cows are housed in shelters as cow slaughter is not legally permitted in most states. The welfare assessment of these old, abandoned, infertile, unproductive and rescued cows was carried out based on the measurement of cow-based and resource-based welfare parameters. The aim of this cross-sectional study was to explore the prevalence of lameness in these sheltered cows and the risk factors associated with it. Fifty-four shelters in six states of India were visited and 1620 cows were clinically examined for lameness and measurements of other animal and resource-based welfare parameters. The prevalence of lameness in these shelters was less than that recorded in dairy cows in conventional Indian farming systems. Lameness was associated with several cow factors: inadequate body conformation, lesions on the hock and carpal joints, dirty udders, overgrown claws and diarrhea. There was evidence of an association with an absence of bedding and a steep gradient of the floor. Improvement in the feeding practices, attention towards cleanliness, and improvements in shelter flooring may reduce the prevalence of lameness and improve cow welfare in the shelters.

**Abstract:**

The sheltering of old, unproductive and abandoned cows in traditional cow shelters, known as gaushalas, has been practiced in India since ancient times. Cows are kept in these shelters until they die of natural causes. The welfare of the cows in these shelters was assessed through a cross-sectional study of 54 cow shelters in six states of India. A total of 1620 cows were examined to assess the prevalence of lameness in these cows, and the associated risk factors for lameness were identified through the measurement of animal-based and resource-based welfare indicators. The overall lameness prevalence was 4.2%. The majority (86%) had mild to moderate hock joint swellings but no or only mild carpal joint injuries. Approximately one-half had mild to moderate hock joint hair loss and most were free of hock joint ulcerations. Claw overgrowth was present in almost one half of the cows. Lameness prevalence was positively correlated with coat dirtiness, hock and carpal joint lesions, diarrhea and claw overgrowth scores. In a multivariate analysis, lameness prevalence increased as the Body Condition Score (BCS) decreased and was associated with increased udder dirtiness, the ulceration of the hock joint, carpal joint injuries and claw overgrowth. Resource-based indicators measured at the shelter level suggested that an absence of bedding in the sheds and an increase in the gradient of the shed flooring increased lameness. Addressing the principle risk factors identified for lameness in the sheltered cows (low body condition, dirty udders, lesions on the hock and carpal joints, overgrown claws, and a steep floor gradient) may help to reduce this serious animal welfare problem.

## 1. Introduction

The sheltering of old, abandoned, unproductive and stray cows in traditional cow shelters, or Gaushalas, is a five-thousand-year-old tradition in India [1]. The cows are housed in shelters until they die of natural causes. The management of these shelters is organized by temples and public trusts, with the financial support of the public, philanthropists, non-governmental organizations and the Indian Government [2]. Animal health care management is a major challenge faced by shelter managers due to the paucity of funds and lack of trained manpower [1,3].

Determining the relationships between husbandry practices and cow health is important to develop protocols for husbandry that will improve welfare [4]. Lameness is a health problem in cows that has significant welfare implications due to the pain induced, the effects on mobility, the long duration of the illness [5] and its greater prevalence in herds all over the world [6,7,8]. Most welfare assessments of cattle herds have lameness as one of the most important animal-based measures in their protocol [9,10,11,12,13]. Prevalence rates of lameness in dairy herds range from 17 to 35% in most parts of the world where it has been measured, including the United Kingdom, Canada, Italy, United States, and Malaysia [7,14,15,16,17,18]. The reported incidence of lameness in dairy cows in India ranges from 8.1 to 30.5% [19,20,21]. Intensively managed systems are particularly associated with lameness in cattle [22]. Known risk factors include lying behavior, hock lesions, limb hygiene, inadequate stall dimensions, insufficient or low-quality bedding, slippery walking surfaces, and exposure of the feet to slurry [23,24,25,26,27,28]. The prevalence of lameness in cow shelters has been reported in our descriptive study [29] and the same data set has been used in this study for further analysis of the association of lameness with other animal- and shelter-based welfare parameters. There is a possibility of such association if the shelters have conditions, such as poor flooring that predispose the cows to lameness. Therefore, the objective of this study was to determine the associated risk factors with lameness in a cross-sectional study of the welfare of cows in shelters.

## 2. Materials and Methods

Cows in 54 shelters (gaushalas) located in the six states of India (Gujarat, Maharashtra, Rajasthan, Punjab, Haryana and Himachal Pradesh) were assessed for their welfare. These six states are located in the northern and western part of India, have the most shelters, and have a tradition of sheltering cows, except one (Himachal Pradesh), which is establishing new shelters to manage the street cow problem. Of the 54 shelters, 26 were visited on the advice of state veterinary officers or the Animal Welfare Board of India (AWBI), and the remaining shelters were chosen using a snowballing technique, taking recommendations from shelter managers. There was no significant difference (*p* < 0.05) between shelters obtained by the two methods in any measured parameter when compared by analysis of variance or a Moods median test (in the case of non-normal residuals). A single 2 d visit was made to each shelter between December 2016 and July 2017. In each shelter, 30 cows were sampled, following a power calculation to determine the required numbers of cows and shelters [30], to detect an odds ratio of 4 with a power of 0.8 and α = 0.05. The sample size of 30 cows was sufficient to estimate within-herd prevalence with an error of 10% at a 95% level of confidence. Cows were selected by choosing every third cow in the shed or the yard, and 1620 cows were sampled in total. 

Data collection included the recording of direct observations of the cows and measurements, as well as the recording of the various housing parameters (resource-based parameters). Management data (feeding time and regime, frequency of water provision to the cows if not available ad libitum, duration of pasture grazing and access to yards, frequency of scraping the floors) were collected in a 30-minute interview of the shelter manager, based on a predesigned questionnaire which is available on request. Twelve animal-based parameters were chosen based on a literature search and our experience of welfare issues in shelters: lameness score, lactation status, cow age, Body Condition Score (BCS), dirtiness of the hind limbs, dirtiness of the udder, dirtiness of the flanks, hock joint swellings, hock joint hair loss, hock joint ulceration, carpal joint injuries and claw overgrowth.

### 2.1. Animal-Based Welfare Parameters

Lameness scoring was undertaken by scoring the locomotion of each sampled cow according to a 5-point scale (Table 1) developed by Sprecher et al. [31]. The lactation status (lactating or non-lactating) of the cows was recorded, and the age of each cow was approximated from the shelter’s records, an interview with the shelter managers and from the cows’ teeth. The BCS was assessed by visual inspection of the cows from the side and back of the cows, with units ranging from 1 (lean) to 5 (fat), scored to quarter points as described by Edmonson et al. [32] and modified by Thomsen and Baadsgaard [33]. Cows with a score of ≤ 1.25 were considered emaciated, 1.5–2 thin, 2.25–3.75 normal and 4 or more obese.

The dirtiness of the hind limbs, udder, flanks and body hair loss was assessed by visual inspection on both sides of the cow and from behind, as described by Whay et al. [13]: 1—no dirtiness; 2—mildly dirty (small soiled areas of dirtiness with no thick scabs); 3—medium dirtiness (large soiled areas but with < 1-cm thick scabs of dung) and 4—severely dirty (large soiled areas with > 1-cm thick dung scabs). The body hair loss score was assessed as: 1—no hair loss, 2—mild hair loss, 3—moderate hair loss, and 4—severe hair loss [13].

The hock region was defined as the lateral tarsus, medial tarsus and the lateral, medial and dorsal calcaneus. Both of the hind limbs of each cow were visually inspected and examined. Hock lesions included hair loss, ulcerations and swellings in a modification of the method of Wechsler et al. [34] and Whay et al. [13]. Hair loss and ulceration on the joints were scored as: 0—no hair loss or ulceration, 1—mild hair loss or ulceration < 2 cm^2^, 2—medium hair loss or ulceration (approximately 2.5 cm^2^), 3—severe hair loss or ulceration > 2.5 cm^2^. Hock joint swellings were scored as: 1—mild swollen joint, 2—medium swollen joint, and 3—severely swollen joint. Carpal joint injuries were scored as: 0—no skin change, 1—hairless, 2—swollen, and 3—with wound [34]. Claw overgrowth was visually inspected on each sampled cow and scored according to the scale devised by Huxley and Whay [35]: 0—normal claw, 1—mild claw overgrowth, 2—moderate claw overgrowth, and 3—severe claw overgrowth.

### 2.2. Resource-Based Welfare Parameters

The area of each shelter shed was calculated after measuring the length and breadth of shed using a laser distance meter (CP-3007 model, ultrasonic distance meter 40 KHz frequency, Chullora, New South Wales, Australia), confirmed by measurement with a measuring tape. The space allowance per cow in the shed was calculated by dividing the area of the shed by the total number of cows housed within that shed. In shelters with cows in tie-stalls, the space allowance was calculated by finding the area covered by a cow in each such stall [36]. In shelters where the cows were tethered but not in stalls, the space allowance was calculated by measuring the length of tether rope from where it was tied to a peg to the hind limb of the cow when fully extended. This allowed calculation of the diameter of a semicircular area in which the cows was able to move. Using the formula for calculating the area of a semi-circle (πr^2^/2), the area per cow was calculated for each tethered cow.

The types of flooring of the sheds and yards were recorded. The Coefficient of Friction (CoF) of the flooring of the shed was determined as the force required to move an object on a floor, divided by the weight of the object [37], using a 1-kg/10 N spring balance attached by a hook to a cuboid wooden block weighing 156 g. This block was gently pulled across the floor at a speed of 0.17 m/second and the minimal frictional force (F) required to keep it moving was recorded [38]. The CoF in the lying areas and passages of the shelter sheds was calculated using the above-mentioned formula. The presence or absence of bedding in the shelter sheds and type of bedding of the sheds was recorded by visual inspection.

The cleanliness levels of the shelters were assessed by estimation of the percentage of floor covered by dung in the lying areas and passages of sheds and yards [39]. Similarly, the proportion of the floor covered with urine in lying areas and the passages of the sheds was visually determined. The average gradient of the flooring in the shed lying areas, passages and yards was recorded at three different places using vertical and horizontal measurements with an inclinometer (Bosch Professional, 600MM, DNM60L Model, Clayton, Australia).

## 3. Statistical Analysis

Descriptive and other statistical analyses were conducted using statistical software—Minitab.17.1.0. (Minitab® version 17.1.0, Minitab Inc., State College, PA, USA). Variables were tested for normality by the Anderson–Darling test [40]. The univariate analysis of cow-based variables for each shelter was conducted using Spearman’s rank correlations, because not all of the variables were found normally distributed by the Anderson–Darling test. This investigated correlations between mean shelter values for lameness and the other cow-based variables, which were continuously distributed.

We then generated two sub-models for the data analysis. In the first, cow-based risk factors for lameness were examined in a multivariate analysis. We then attempted ordinal regression modeling using all five lameness scores as outcome variables, but the models did not show a biologically plausible association between lameness and predictors. This was because we had very few cows with scores of 4 or 5. Hence lameness scores were transformed into binary values, cows that were clinically not lame (0), and scores of 3, 4 and 5 as clinically lame cows (1). A binary logistic regression analysis with the logit procedure and the modeled outcome lameness (present or not), based on the locomotion score of the sampled cows, was undertaken. Predictor variables (dirty hind limbs, udder, flanks, body hair loss, tarsal joint swellings, tarsal joint hair loss, tarsal joint ulceration, carpal joint injuries, diarrhea and claw overgrowth) were also dichotomized by classifying them as the absence of a lesion/change (scores 0 and 1) or the presence of a lesion (scores 2, 3 and 4, as prescribed by the scoring system of the variable). Thus, these dichotomous variables were defined as 0 or 1, with 1 representing the expected increased risk. Observations within shelters were accounted for by including shelter as a clustering effect in the model. The residuals were analyzed to explore the basic assumptions of logistic regression and model fit, according to Dohoo et al. [41]. The graphical examination of the residuals showed them to be normally distributed. Levels of significance were set as *p* ≤ 0.05 for all analyses.

In the second sub-model, resource-based and management parameters were analyzed at the shelter level. Lameness prevalence estimates at the shelter level were used as the outcome in analyzing risk factors. The multivariate analysis of the effects of lameness on resource-based parameters was performed by a Stepwise General Linear Model (GLM) with α to enter at 0.15. The residuals were normally distributed (*p* = 0.27) but were also examined graphically.

## 4. Results

### 4.1. Animal-Based Welfare Parameters

Categorical animal-based parameters are enumerated in Table 2. The overall prevalence of lameness in the cow shelters was 4.2%. Out of the 1620 cows examined, only 69 cows were clinically lame (locomotion scores 3 to 5; 3–3.2%, n = 53, 4–0.9%, n = 15, and 5–0.06%, n = 1). Most (n = 1373, 84.7%) of the cows were not lame (score 1), and 11% (n = 178) of the cows had mild/subclinical lameness (score 2).

The median age of the cows in the shelters was 11 years (Q_1_ = 8, Q_3_ = 14 years; Inter Quartile Range (IQR) = 6 years) and the majority were non-lactating (87.9%, n = 1425). The median BCS was 2.75 (Q_1_ = 2.25 and Q_3_ = 3.25; IQR = 1.0), most cows being in the normal range for the BCS, i.e., 2.25 to 3.75 (75.4%, n = 1233). Some were thin (BCS range of 1.5 to 2, 22.8%, n = 371) and very few were obese (BCS 4 and above, 1.4%, n = 24).

Most cows had mild to moderately dirty (scores 2 and 3) hind limbs (85.6%, n = 1387), udder (75.9%, n = 1231) and flanks (74.2%, n = 1203). Almost half of the cows had no body hair loss (score 1, 45%, n = 728) and the rest had just mild to moderate hair loss (scores 2 and 3) (53.2%, n = 863). Hock joint swellings and hair loss were mostly mild or moderate (swellings 86%, n = 1394; hair loss 76.6%, n = 1243). Hock joint ulceration was mostly absent (score 0, 53.6%, n = 869), but mild ulceration (score 1, 33.2%, n = 539) was common and moderate ulceration occasional (score 2, 12.9%, n = 210). Carpal joint injuries were mostly either absent (score 0) or mild (score 1) (total 86.6%, n = 124). Claw overgrowth was absent (score 0) in 52.4% of the cows (n = 850), but mild overgrowth was common (36.3%, n = 589) and moderate or severe claw overgrowth levels (scores 2 and 3) occasionally observed (11%, n = 181). Diarrhea was observed (score 1) in 4.2% of the cows (n = 69).

#### Relationship between Lameness and Animal-Based Measures

The univariate analysis of the animal-based welfare measures by Spearman’s rank correlation found significant (*p* < 0.05) positive correlations of lameness with dirtiness of hind limbs, udder, and flanks, and also with body hair loss, carpal joint injuries, diarrhea, claw overgrowth, cow age and hock joint swelling, hair loss and ulceration (Table 3).

In the multivariate analysis, lameness, as a binary outcome variable, was related with the BCS of the cows, udder dirtiness, hock joint ulceration, carpal joint injuries and claw overgrowth (Table 4). Lame cows were associated with a low BCS (OR = 0.64, CI = 0.42–0.97), but lameness was increased in cows with dirty udders (OR = 2.13, CI = 1.25–3.61), tarsal joint ulcerations (OR = 2.54, CI = 1.10–5.84), carpal joint injuries (OR = 3.75, CI = 1.81–7.75) or overgrown claws (OR = 2.67, CI = 1.50–4.73).

### 4.2. Shelter and Resource-Based Welfare Parameters at the Shelter Level

The median space availabilities provided for the cows in the sheds and yards were 2.73 and 5.90 m^2^/cow, respectively. We found four types of floors in the shelters—earth, brick, stone and concrete. Concrete floors were the most predominant (42 sheds), followed by earth (21 sheds), brick (19 sheds) and stone (4 sheds). The floors of the yards were predominantly earth (41 yards), followed by concrete (19 yards), brick (13 yards) and stone (3 yards). The median CoF of the shed flooring was 0.43. In 96% of the shelters, no bedding was provided. The median percentages of dung present in the lying areas and passages of the shed were 15 and 10%, respectively. The median percentage of dung in the yards was 20%. The average floor gradient in the shed lying area, shed passage and shelter yard was 1.46, 2.36 and 1.51, respectively (Table 5). In 83.3% (45 shelters) of the lying areas and passages of the sheds, urine was not found accumulated on any part of the floors. In 89% of the yards (48 shelters), there was no accumulation of urine on the floors. The median values of duration of access to yards and pastures were 8 hours/d and zero. Shelter yards were usually cleaned twice in a day.

The cows in all shelters were offered a basal feed of straw (mean 17.6 kg/cow/day), either thrice or twice daily, from locally available crops (paddy, wheat or millet). Ten shelters (18.5%) fed only straw, but most fed supplements (11 shelters, 20.3%, agricultural byproducts; 25 shelters, 46.3%, agricultural by-products and hay, and 8 shelters, 14.8%, fresh green fodder, typically lucerne, clover, or vegetable waste). In addition, concentrate feeding (grains, flour and rice or wheat husks) were offered at 0.1–0.5 kg/cow in most shelters (43, 85%).

#### Relationship between Lameness and Resource-Based Measures

The univariate analysis of the resource-based welfare measures at the shelter level using Spearman’s rank correlation revealed no relationship (*p* > 0.05) with lameness. In the multivariate analysis model (r^2^ adjusted = 34.1%; residuals were normally distributed, *p* = 0.10), lameness had a significant positive association with the presence of bedding (F = 12.4; *p* = 0.001) and a positive association with the gradient of the shed passages (F = 5.5; *p* = 0.02). The relationship was described by the equation:

Lameness = c + 1.26 (± 0.072) + 0.028 (± 0.012) gradient of shed passages(1)

where the intercept, c, was 1.06 for no bedding and 1.46 for bedding. 

Further exploration of relevant correlations revealed that the hock lesions were negatively correlated with the % dung in the passages (correlation coefficient = −0.283, *p* = 0.04). 

## 5. Discussion

The prevalence of lameness in the present study was 4.2% in the cow shelters, less than reported in dairy herds in the US and North America [16,42], Finland [43], Germany [44] and Norway [45]. Studies on the prevalence of lameness in dairy cows in India are scant and restricted to individual farms, with prevalence levels of 10 and 33% clinical lameness reported in two cross bred herds of 110 and 251 cows, respectively [21,46]. The low prevalence of lameness in sheltered Indian cows could be attributed to the absence of production stress which would arise from the commercial use of the cows for milk and the very limited energy-rich concentrate feeding [38]. In 85% of the shelters recorded in this study, a very insufficient quantity of concentrate diet (<0.5 kg/cow) was fed to the cows.

Differences in lameness prevalence rates could also be due to housing and management conditions, lameness scoring methods and threshold scores used, as well as breed differences in lameness susceptibility [43].

The median age of the cows in the shelters was 11 years, which demonstrates that it was an older age group than commercial herds, and some lameness may be explained by the long exposure of some cows to the shelter housing and flooring. However, although age has been reported as a risk factor for lameness in dairy cows [16,43,47], it was not a significant factor in the multivariable model for lameness prevalence at the shelter level in this study.

The strong association between a low BCS and lameness in our study corroborates the findings of previous authors [16,17,48,49,50]. A low BCS in cows is both phenotypically and genetically positively associated with susceptibility to lameness [15,51]. Lameness leads to reduced movement (including potentially to feed and water supply), a slower feeding rate and decreased feed intake, all of which potentially reduce the body condition of the cows [16,52,53]. The lack of movement is partly due to a reduced digital cushion (a fatty pad located in the claw capsule), which serves as a shock absorber to the third phalanx when it bears the weight of the cow during the interaction of the hoof with the flooring [54]. However, this digital cushion is much reduced in cows with a low BCS, increasing susceptibility to lameness [55], indicating a bidirectional relationship. Lame cows in the shelters may arrive late at the feed bunks, where the leftover feed is restricted in quantity and quality. This effect of lameness on the BCS in the sheltered cows may be more profound than for dairy cows, as the shelter feed, being for subsistence only, is of low quality. Cows with a low BCS are also more susceptible to lameness due to non-infectious lesions of the feet [56]. Furthermore, a low BCS may predispose cows to body lesions; higher BCS cows have fewer protruding bones which has a protective effect against tarsal lesions [48,57].

The dirtiness of the udder in the shelter cows was another risk factor for lameness. This could be attributed to the associated dirtiness of the floor, mainly with slurry which soils the udder and limbs while a cow is lying, standing and walking. Dirty conditions are known to predispose cows to lameness [7]. Poor hygiene in terms of the accumulation of dung and urine in the lying areas and passages predisposes the hooves to various lesions leading to lameness [58,59]. The median of 15, 10 and 20% of the floors of the lying areas, passages and yards of the shelters, respectively, covered with dung (Table 5) signifies an increased level of dirtiness in these areas. A previous study [39] investigated three husbandry systems (tie stalls with seasonal outdoor access, tie stalls with daily outdoor access and loose housing with daily outdoor access) for floor dirtiness, using the % of dung in lying areas as the main measure. Their findings of 11–17% dung in the three different housing systems are similar to our results (median 15% dung in the lying area), which was probably because we also had tie stalls and loose housing with variable outdoor access.

The presence of hock joint ulcerations and carpal joint injuries as risk factors for lameness could be due to the type of flooring surfaces of the shelter premises. Tarsal joint lesions arise from (1) the abrasiveness of the floor [47]; (2) the continuous increased pressure on the limbs and body from the body weight of the cows and the inelastic flooring surface affecting the blood circulation to these areas [60]; and (3) the collision with the flooring surface when getting up and lying down. In our study, the median CoF of the flooring in the shelters was 0.43, which shows that the floors were not very abrasive and there was a vulnerability of the cows to slipping. This CoF value is close to the critical value of 0.4, below which there is an exponential increase in the risk of slipping [37,61]. This indicates the floor surface lacked adequate friction, perhaps because of the old age of some shelters, with repeated wear. Hock and knee joint lesions are also attributed to the type and condition of the flooring surface of the housing [35,39,62,63]. Lesions on the hock joints have been implicated in the causation of lameness in cows [13,48]. Lame cows experience difficulties in lying down or getting up, leading to the hock and carpal areas getting abraded on the rough floors of the shelters. There is a possibility that the hock and carpal lesions could be painful, resulting in lameness, but the possible direction of causation cannot be determined in a cross-sectional study [17].

The small area per cow, absence of bedding in most cow shelters and presence of slurry in the shelter premises found in this study could have contributed to the presence of hock joint lesions in the cows. As the hock lesions were negatively correlated with the % dung in the passages, we hypothesize that the dung may protect the cows lying in the passage from contact with the rough floors. An examination of the effects of dung in increasing slipping or protecting from contact with rough floors is worthy of further study. The presence of bedding prevents abrasions on the limbs and, if absent, as was the case for 96% of shelters in our study, a hard floor may impede circulation [64]. Low space allowance, slurry laden floors and abrasive concrete floors have been identified as risk factors for limb lesions and lameness in dairy cows [47,64,65,66]. The typical lying down and getting up behavior of the cows, in which both the knee joints touch the floor surface explains the injuries on the knee joints, due to the constant abrasions on rough floors causing lameness [48], and the third quartile CoF of shelter floors in our study was 0.65, which represents quite abrasive/rough floors. Approximately half of the shed floors (42%) of the shelters were made of concrete, which is hard and sometimes abrasive [48], leading to lesions on the joints, and increased susceptibility to lameness [67].

Claw overgrowth was a risk factor for lameness in our study, which concurs with previous studies [67,68]. According to a review by Ter Wee et al. [69], 90% of lameness problems in cattle are due to claw abnormalities. Claw overgrowth changes the claw confirmation, which is associated with lameness [70,71]. It often results from an increased rate of horn growth, associated with laminitis, sole ulcers and white zone lesions in cows [72,73,74]. Walking on the hard surfaces found in some shelters could lead to biomechanical injuries to the claws due to the reaction of the forces from the hard floor at the point of interaction of the claw and floor. The consequent overgrowth of the claws, especially the outer ones, predisposes the animal to pathological lesions [75]. Additionally, the slurry in the lying areas and passages wets the floor and keeps the cow hooves continuously wet. This leads to claw overgrowth, irregular weight bearing sole surfaces, claw injuries and disruption of claw horns [17,76,77]. This etiology supports the claw overgrowth leading to lameness in our study, as concrete flooring and the presence of dung on the floors were found in most shelters.

The presence of urine in the shelter passages increases slurry formation, and the constant wetness of the foot in the slurry can erode the soft heel bulb [78], a risk factor for lameness. Furthermore, slurry and wet floors increase floor slipperiness, which predisposes cows to the risk of injuries due to slipping, and the resultant dirtiness of feet and legs causes conformational changes of the claw, also predisposing to lameness [17,79]. The slope of the floors is a risk factor for laminitis in dairy cows [80].

The absence of bedding in most of the sheds in our study, albeit with a small sample size, might be a risk factor for lameness in the shelter cows. Cows prefer bedded floor surfaces for lying and standing [81]. The comforting cushioning effect of the bedding while standing, and the increased lying times on bedded floors have potential benefits against lameness. The presence of bedding decreases the prevalence of lameness in dairy farms [22,82].

A significant association of lameness with the gradient of the shed passages in our study might be due to discomfort during lying, leading to more standing on the floors, restlessness, increased muscle activity and possible fatigue [83]. Hock swellings are a cause of lameness in dairy cows and have been observed to increase with gradient of the stall floors [47]. An increase in floor gradient may increase the risk of slipping and consequent hock lesions in the form of abrasions and swellings. However, another study has shown that prolonged standing time on a sloped floor (a 5% slope) improved claw health because it allowed better drainage and reduced hoof contact with excreta [84]. The floor gradient should be adequate to provide drainage without contributing to lameness and limb injuries. Therefore, the proper design of the cowsheds may reduce lameness and associated lesions on the limbs of the cows.

The strength of our study lies in a large number of cows and shelters assessed, producing a comprehensive set of animal- and resource-based welfare parameters in a unique context in which cows are not yielding milk. The cross-sectional study revealed numerous associations but inferences about causality are limited. 

## 6. Conclusions

The prevalence of lameness in the cows in the shelters was less than has usually been recorded for cows in dairy farms. The risk factors for lameness in the sheltered cows that we identified were inadequate cleaning of the premises, improper flooring and probably a lack of a balanced feeding regimen. These shortcomings in the management of the shelters have manifested in the form of the reduced body conditions of the cows, dirty udders, dirty limbs, lesions on the hock and carpal joints and the overgrowth of claws, which were risk factors for lameness. Improvement in these aspects will improve cow welfare by reducing the prevalence of lameness. The shelter cleanliness of the shelter premises by the elimination of slurry in the lying areas and the passages will promote better foot hygiene. The provision of bedding in lying areas reduces hock lesions and standing times, and provides comfort [34], ultimately reducing lameness. The flooring of the cow shelters should be improved as many had concrete flooring that was hard and rough, or slippery in the absence of bedding. The flooring is implicated as a major cause of lesions in the limbs and joints. Sand as a bedding material can be considered as an option for a softer lying area, though labor costs involved should be accounted for. Further work is required on the effect of floor slope on lameness taking into consideration the flooring material characteristics. Good feeding management is very important to maintain good body condition in the retired and abandoned cows in the shelters, as a low BCS risks the cows developing lesions on the hock and carpal joints, predisposing them to lameness, as well as compromising the general health of the cows. Improving the managerial aspects in terms of cleanliness, feeding and floor comfort will reduce lameness and lead to the better welfare of the cows in the shelters.

## Figures and Tables

**Table 1:** Lameness Scoring System used in the study to determine the prevalence of lameness ^a^.

Locomotion Score	Interpretation	Description of Locomotion
1	Normal	Normal walk with a flat back
2	Mild lameness	Normal walk but with an arched back
3	Moderate lameness	Slight abnormal walk, short stride with one or more legs
4	Lameness	Visibly lame, but able to bear some weight on all legs
5	Severe lameness	Almost complete transfer of weight from an affected leg

^a^ Adopted from Sprecher et al. [30].

**Table 2:** Percentage of cows in each category (number) of animal-based welfare parameters in 54 shelters (n = 1620 cows), see text for details of scoring systems.

Parameter	Score 0	Score 1	Score 2	Score 3	Score 4	Score 5
Lameness score (scale 1–5)	-	84.7 (1373)	10.9 (178)	3.2 (53)	0.9 (15)	0.06 (1)
Lactation status (scale 0, non-lactating, 1, lactating)	87.9 (1425)	12.04 (195)	-	-	-	-
Dirty hind limbs score (scale 0–3)	2.3 (38)	42.5 (690)	43.02 (697)	12.04 (195)	-	-
Dirty udder score (scale 0–3)	17.4 (283)	44.5 (722)	31.4 (509)	6.5 (106)	-	-
Dirty flanks score (scale 0–3)	19.5 (316)	42.2 (684)	32.0 (519)	6.2 (101)	-	-
Body hair loss score (scale 0–3)	44.9 (728)	30.3 (492)	22.9 (371)	1.7 (29)	-	-
Body Condition Score (BCS) (scale 1–5) 1(≤1.25) 2(1.5–2) 3(2.25–3.75) 4(4 and above)	-	0.1 (2)	22.8 (371)	75.4 (1223)	1.4 (24)	
Hock joint swelling score (scale 0–3)	11.7 (191)	22.3 (262)	63.7 (1032)	2.1 (35)	-	-
Hock joint hair loss score (scale 0–3)	22.9 (372)	49.3 (800)	27.3 (443)	0.3 (5)	-	-
Hock joint ulceration score (scale 0–3)	53.6 (869)	33.2 (539)	12.9 (210)	0.1 (2)	-	-
Carpal joint injuries score (scale 0–3)	44.8 (726)	31.8 (516)	23.0 (373)	0.3 (5)	-	-
Claw overgrowth score (scale 0–3)	52.4 (850)	36.3 (589)	9.6 (156)	1.5 (25)		
Diarrhea (scale 0–1)	95.7 (1551)	4.3 (69)	-	-	-	-

**Table 3:** Significant (*p* < 0.05) Spearman’s rank correlations between lameness (scores from 1 (not lame) to 5 (severely lame)) and other animal-based variables.

Variables	Correlation Coefficient	*p*-Value
Age (years)	0.099	≤0.001
Dirty hind limbs score (scale 0–3)	0.147	≤0.001
Dirty udder score (scale 0–3)	0.160	≤0.001
Dirty flanks score (scale 0–3)	0.188	≤0.001
Body hair loss score (scale 0–3)	0.060	0.015
Hock joint swelling score (scale 0–3)	0.064	0.010
Hock joint hair loss score (scale 0–3)	0.051	0.040
Hock joint ulceration score (scale 0–3)	0.092	≤0.001
Carpal joint injuries score (scale 0–3)	0.223	≤0.001
Diarrhea score (scale 0–1)	0.112	≤0.001
Claw overgrowth score (scale 0–3)	0.360	≤0.001

**Table 4:** Binary logistic regression of lameness with other animal-based welfare parameters in shelter cows (n = 1620).

Parameter/Variable	Coefficient	Odds Ratio (OR)	Confidence Interval (CI)	*p*-Value
Constant	−3.974	-	-	≤0.001
Body Condition Score (BCS)	−0.444	0.64	0.42–0.97	0.03
Dirty udder	0.758	2.13	1.25–3.61	0.004
Hock joint ulceration	0.934	2.54	1.10–5.84	0.04
Carpal joint injuries	1.322	3.75	1.81–7.75	<0.001
Claw overgrowth	0.983	2.67	1.50–4.73	<0.001

**Table 5:** Descriptive statistics of resource-based welfare parameters of cow shelters (n = 54).

Parameter	1st Quartile (Q_1_)	Median	Third Quartile (Q_3_)	Inter Quartile Range (IQR)
Shed area per cow (m^2^/cow)	1.56	2.73	3.62	2.06
Yard area per cow (m^2^/cow)	3.60	5.90	21.50	17.90
Coefficient of friction of shed flooring (CoF)	0.27	0.43	0.65	0.37
Percentage of dung present in lying areas of the shed	5.00	15.00	40.00	35.00
Percentage of dung present in passages of the shed	5.00	10.00	42.50	37.50
Percentage of dung present in the yards	10.0	20.0	40.0	30.0
Average gradient of the flooring of lying areas of the shed (%)	0.96	1.46	2.20	1.23
Average gradient of the flooring of passages of the shed (%)	1.27	2.36	3.52	2.24
Average floor gradient in yards (%)	1.13	1.51	2.43	1.30
Duration of access to pasture (h/d)	0.0	0.0	6.0	6.0
Duration of access to yard (h/d)	4.0	8.0	24.0	20.0
Frequency of cleaning of yards (number of times/d)	1.0	2.0	2.0	1.0
